# Migraine predicts physical and pain symptoms among psychiatric outpatients

**DOI:** 10.1186/1129-2377-14-19

**Published:** 2013-02-27

**Authors:** Ching-I Hung, Chia-Yih Liu, Shuu-Jiun Wang

**Affiliations:** 1 Department of Psychiatry, Chang-Gung Memorial Hospital at Linkou and Chang-Gung University School of Medicine, Taoyuan, Taiwan; 2 Neurological Institute, Taipei Veterans General Hospital and National Yang-Ming University School of Medicine, No. 201 Shi-Pai Road, Section 2, Taipei 112, Taiwan

**Keywords:** Depression, Anxiety, Headache, Pain, Quality of life, Somatization

## Abstract

**Background:**

No study has been performed to compare the impacts of migraine and major depressive episode (MDE) on depression, anxiety and somatic symptoms, and health-related quality of life (HRQoL) among psychiatric outpatients. The aim of this study was to investigate the above issue.

**Methods:**

This study enrolled consecutive psychiatric outpatients with mood and/or anxiety disorders who undertook a first visit to a medical center. Migraine was diagnosed according to the *International Classification of Headache Disorders*, 2^nd^ edition. Three psychometric scales and the Short-Form 36 were administered. General linear models were used to estimate the difference in scores contributed by either migraine or MDE. Multiple linear regressions were employed to compare the variance of these scores explained by migraine or MDE.

**Results:**

Among 214 enrolled participants, 35.0% had migraine. Bipolar II disorder patients (70.0%) had the highest percentage of migraine, followed by major depressive disorder (49.1%) and only anxiety disorder (24.5%). Patients with migraine had worse depression, anxiety, and somatic symptoms and lower SF-36 scores than those without. The estimated differences in the scores of physical functioning, bodily pain, and somatic symptoms contributed by migraine were not lower than those contributed by MDE. The regression model demonstrated the variance explained by migraine was significantly greater than that explained by MDE in physical and pain symptoms.

**Conclusions:**

Migraine was common and the impact of migraine on physical and pain symptoms was greater than MDE among psychiatric outpatients. Integration of treatment strategies for migraine into psychiatric treatment plans should be considered.

## Background

Migraine, mood disorders, and anxiety disorders are comorbid with each other and interact [1-6]. Many studies have investigated the prevalence and impact of depression and/or anxiety among patients with migraine. These studies show that comorbidity with depression and/or anxiety is common and related to a poorer quality of life, increases the suicide risk, and predicts a poorer outcome among patients with migraine [7-11].

Many other studies, on the contrary, have investigated the frequency and impact of migraine among patients with mood and/or anxiety disorders [12-20]. Most of these studies evaluated the impact of migraine on psychiatric outpatients with depression or major depressive disorder (MDD) [12-16]. Comorbidity with migraine is associated with more severe depression, anxiety, and somatic symptoms, as well as a poorer health-related quality of life (HRQoL) [14-16].

Patients with depression may suffer from impaired multiple functions and have an increased social burden [21]. Major depressive episode (MDE) is one of the worst states of mood disorders and may be most able to reflect functional impairments of depression. Similarly, migraine also causes significant functional impairment in multiple dimensions [22]. To the best of our knowledge, no study has been performed to compare the impacts of migraine and MDE on depression, anxiety and somatic symptoms as well as HRQoL among psychiatric outpatients with mood and anxiety disorders (or non-psychotic outpatients).

Understanding the impact of migraine among psychiatric outpatients with mood and/or anxiety disorder is important because the information might alert physicians to note migraine and to integrate the treatment plan for mood disorders, anxiety, and migraine. Therefore, the study aimed to compare the differences in depression, anxiety and somatic symptoms, as well as HRQoL, associated with the presence of migraine or MDE among psychiatric outpatients with mood and/or anxiety disorders. We hypothesized that psychiatric outpatients with migraine have a greater severity of depression, anxiety, and somatic symptoms as well as a poorer HRQoL based on previous studies [12-16]. Moreover, the impacts of migraine on physical and pain symptoms might be as important as those of a current MDE.

## Methods

### Subjects

This study enrolled subjects between September 2007 and August 2009 in the psychiatric outpatient clinic of Chang Gung Memorial Hospital, a medical center in northern Taiwan. The study participants were consecutive psychiatric outpatients (20–60-years-old) who visited our clinic for the first time and had not taken any medications for psychiatric disorders and migraine prevention within the previous 4 weeks. Four exclusion criteria were established: 1) psychotic disorders, such as schizophrenia, delusional disorder, and other psychotic disorders; 2) mental retardation, delirium, dementia, and mental disorders due to general medical conditions; 3) psychotic symptoms, catatonic features, severe psychomotor retardation, or a current manic episode in the index month, which may cause difficulty in completing self-administered scales or cooperating with the study process; 4) a history of substance dependence or abuse without full remission in the index month.

The government-run single-payer compulsory National Health Insurance program in Taiwan enrolls more than 96% of Taiwan’s population [23]. The insured in Taiwan have complete freedom to choose their health care providers. They can seek care at tertiary care institutions, regardless of the severity of their illness. Only 2.3% of patients in the clinics of a university teaching hospital in Taiwan were referred from primary clinics [24]. Therefore, although our patients were collected from a medical center, they did not differ much in the demographic patterns of those attending primary psychiatric clinics.

The study was approved by the Institutional Review Board of Chang Gung Memorial Hospital. A written informed consent, based on the guidelines regulated in the Declaration of Helsinki, was obtained from all subjects prior to study enrollment.

### Assessment of the severity of depression, anxiety, and somatic symptoms, and HRQoL

The Hamilton Depression Rating Scale (HAMD), the Depression and Somatic Symptoms Scale (DSSS), and the Hospital Anxiety and Depression Scale (HADS) were used to evaluate depression, anxiety, and somatic symptoms over the previous week [25-29].

The DSSS is a self-administered scale composed of 12 items in the Depression Subscale (DS) and 10 items in the Somatic Subscale (SS), which includes five pain symptoms and five non-pain somatic symptoms. The reliability and validity of the DSSS have been reported in previous studies [25-27].

The HADS comprises 7 items in the anxiety subscale (HADS-A) and 7 items in the depression subscale (HADS-D) and does not include any somatic symptoms [29]. Therefore, the HADS can measure depression and anxiety without being confounded by somatic symptoms.

The three scales were used simultaneously to investigate the severity of depression, anxiety, and somatic symptoms. The total scores for each scale or subscale range from 0 to 36 for the DS and 0 to 30 for the SS, 0 to 52 for the HAMD, and 0 to 21 for the HADS-D and HADS-A. A higher score indicates a greater severity.

The acute version of the Short-Form 36 (SF-36), which evaluated the HRQoL in the past week with the same items as the SF-36, was used to be compatible with the evaluating period of the three psychometric scales. The SF-36 includes eight domains: physical functioning (PF), role limitations-physical (RP), bodily pain (BP), general health perceptions (GH), vitality (VT), social functioning (SF), role limitations-emotional (RE), and mental health (MH) [30]. A higher score indicates a better HRQoL. The scores of each subscale ranged from 0 (lowest well-being) to 100 (highest well-being). According to the SF-36 Health Survey Manual, a difference of more than 5 points in each subscale of the SF-36 is considered clinically significant. The Taiwanese version of the SF-36 shows good validity and reliability [31].

The DSSS, HADS, SF-36 were self-administered scales. The HAMD was assessed by a psychiatrist, who was blinded to the results of other psychometric scales, psychiatric diagnoses, and headache diagnoses.

### Diagnoses of psychiatric disorders

Another psychiatrist, who was blinded to the diagnosis of headache and the results of these scales, used the Structured Clinical Interview for DSM-IV-TR to diagnose mood and anxiety disorders [32]. If patients had mood and anxiety disorders simultaneously, their diagnoses were categorized as bipolar disorders or MDD, not anxiety disorders. Therefore, this study divided patients into bipolar disorders, MDD, anxiety-only disorders, and others.

### Assessment of headache

#### Headache diagnosis

All patients completed a structured headache intake form, designed to meet the operational criteria of the International Classification of Headache Disorders, 2nd edition (ICHD-2) [33] including information for the diagnoses of migraine and other common headaches, such as headache frequency, intensity, features, aura, locations, duration, and precipitating factors, as well as the amount and frequency of use of drugs taken for pain. An experienced headache specialist (neurologist), who was blinded to the results of the psychiatric diagnoses and psychometric scales, interviewed all patients after they had completed the headache intake form and made headache diagnoses based on the ICHD-2. Patients with chronic migraine (CM) with or without medication-overuse headache (MOH) and episodic migraine (EM) (i.e. migraine with or without aura) were categorized as the migraine group.

In order to understand the synergic effects of a current MDE and migraine on depression, anxiety, and somatic symptoms, patients were divided into four groups: a current MDE with migraine, a current MDE without migraine, migraine without a current MDE, and others (neither a current MDE nor migraine).

#### Headache intensity and frequency assessment

Subjects evaluated their average headache intensity during the previous week using a visual analog scale, with 0 representing “no headache” and 10 representing “headache as severe as I can imagine.” The total number of headache days in the past week was also collected.

### Statistical methods

We used SPSS for Windows 12.0 to perform the statistical analyses. In performing independent *t*-tests, Bonferroni corrections were used due to multiple comparisons (statistical significance: *p* < 0.01 for HAMD, DS, SS, HADS-D, and HADS-A and *p* < 0.006 for the eight SF-36 subscales). One-way analysis of variance (ANOVA) with *post*-*hoc* comparisons by Scheffe’s method was used to test the differences in continuous variables among groups. The Chi-square and Pearson’s correlation tests were used in appropriate situations.

To compare the impacts of migraine and MDE, multiple linear regression analyses with forward selection, which could prevent multicolinearity, were performed. The dependent variables were the scores of the depression, anxiety, and somatic symptoms, as well as the SF-36 subscales. All these dependent variables were continuous variables. Therefore, using multiple linear regression analyses was appropriate. The independent variables included migraine, a current MDE, and five demographic variables (age, gender, educational level, marital status, and employment). Moreover, general linear models were used to calculate the estimated difference contributed by either migraine or MDE after controlling for demographic variables. In statistical analyses, a two-tailed *p* value < 0.05 was considered statistically significant.

## Results

### Subjects

During the study period, 231 patients fulfilled the criteria for enrollment. Of them, 17 patients refused to participate and 214 (92.6%) subjects completed the study. Table 1 demonstrates that 35.0% of these patients had migraine and 15.9% had chronic daily headache (CM with or without MOH or chronic tension-type headache).

**Table 1:** The percentages of headache diagnoses

***Headache diagnosis***	***Number***	***Percentage***
Chronic migraine ^a^	30	**14.0**
Episodic migraine ^b^	45	**21.0**
Probable migraine	29	**13.6**
Chronic tension-type headache	4	**1.9**
Episodic tension-type headache	40	**18.7**
Probable tension-type headache	26	**12.1**
Other headache	12	**5.6**
No headache	28	**13.1**
Total	214	**100**

^a^ Eight (26.7%) patients also had medication-overuse headache.

^b^ Six (13.3%) of them had migraine with aura.

Table 2 shows the percentages of migraine among psychiatric diagnoses. Patients with bipolar II disorder had the highest percentage of migraine (70.0%), followed by MDD (49.1%), only anxiety disorder, and no mood and/or anxiety disorders. Table 3 shows the demographic variables and demonstrates that female patients had a significantly higher percentage of migraine. There was no significant difference in age, educational years, employment, and marital status between patients with and without migraine.

**Table 2:** The percentage of patients with migraine by individual psychiatric diagnoses

	***Number***	***Percentage***
Bipolar II disorder	7/10	**70.0**
Major depressive disorder	54/110	**49.1**
Only anxiety disorder	12/49	**24.5**
No above diagnosis	2 /45	**4.4**
Total	75/214	**35.0**

**Table 3:** Comparison of depression, anxiety, and somatic symptoms as well as demographic variables between outpatients with and without migraine and MDE

	***Total (n = 214)***		***Yes***	***No***	***Estimated difference ***^**a**^
HAMD	18.3 ± 6.3	Migraine (*n* = 75)	21.6 ± 5.5**	16.5 ± 6.1	4.7**
		MDE (*n* = 95)	23.0 ± 4.1**	14.5 ± 5.3	8.1**
DS	18.9 ± 8.6	Migraine	24.1 ± 7.9**	16.1 ± 7.6	7.3**
		MDE	25.3 ± 6.0**	13.8 ± 6.7	10.9**
SS	13.0 ± 7.0	Migraine	17.5 ± 6.7**	10.5 ± 5.9	6.2**
		MDE	16.1 ± 7.1**	10.4 ± 5.9	5.3**
HADS-D	10.3 ± 5.0	Migraine	12.8 ± 4.5**	8.9 ± 4.7	3.8**
		MDE	13.7 ± 3.7**	7.5 ± 4.0	6.1**
HADS-A	12.1 ± 4.4	Migraine	13.9 ± 4.0**	11.2 ± 4.4	2.6**
		MDE	14.4 ± 3.5**	10.3 ± 4.3	4.0**
Age (years)	38.3 ± 10.5	Migraine	38.1 ± 9.6	38.5 ± 10.9	
		MDE	36.3 ± 10.0*	40.0 ± 10.6	
Educational years	12.5 ± 2.9	Migraine	12.2 ± 2.9	12.7 ± 3.0	
		MDE	12.5 ± 2.7	12.5 ± 3.1	
Gender (female, %)	63.1	Migraine	82.7**	52.5	
		MDE	67.4	59.7	
Employed (yes, %)	66.4	Migraine	60.0	69.8	
		MDE	65.3	67.2	
Married (yes, %)	56.5	Migraine	56.0	56.8	
		MDE	47.4*	63.9	

HAMD Hamilton Depression Rating Scale, DS depression subscale of the Depression and Somatic Symptoms Scale (DSSS), SS somatic subscale of the DSSS, HADS-D depression subscale of the Hospital Anxiety and Depression Scale (HADS), HADS-A anxiety subscale of the HADS, MDE a current major depressive episode.

^a^Estimated difference contributed by migraine or a MDE after controlling for five demographic variables. * *p* < 0.05; ** *p* < 0.01.

Patients with migraine was associated with a higher risk of being in a current MDE (69.3% *vs.* 30.9%, odds ratio (OR) = 5.05, 95% CI = 2.75–9.28, *p* < 0.001) compared to those without migraine. Similarly, patients with migraine also had a higher risk of a past suicide attempt history than those without (30.7% *vs.* 13.7%, OR = 2.79, 95% CI = 1.40–5.57, *p* < 0.01).

### Comparisons of depression, anxiety, and somatic severities between patients with and without migraine

Table 3 shows the severities of depression, anxiety, and somatic symptoms between patients with and without migraine and with and without a current MDE. Compared to patients without migraine, those with migraine had a significantly (*p* < 0.01) greater severity of depression (HAMD, HADS-D, DS), anxiety (HADS-A), and somatic symptoms (SS). Compared to patients without a current MDE, patients with a current MDE also had a significantly greater severity of depression, anxiety, and somatic symptoms. After controlling for demographic variables by a general linear model, the estimated difference in the SS score contributed by migraine was slightly greater than that contributed by a current MDE (6.2 *vs.* 5.3).

**Figure 1:**
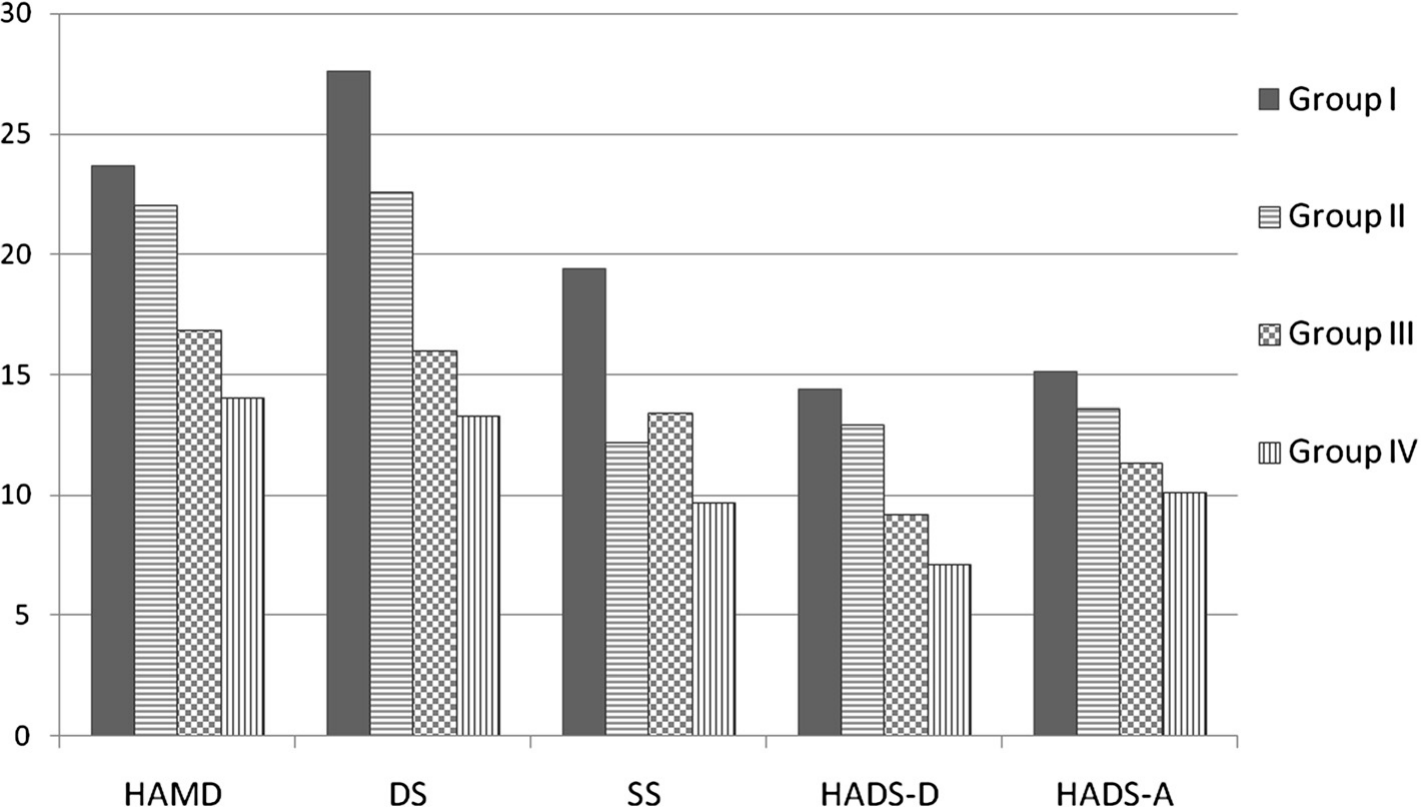
**The severities of depression, anxiety, and somatic symptoms among patients with different diagnoses or comorbidities.** Group I = a current major depressive episode (MDE) comorbid with migraine; Group II = a current MDE without migraine; Group III = migraine without a current MDE; Group IV = neither a current MDE nor migraine. HAMD Hamilton Depression Rating Scale, DS depression subscale of the Depression and Somatic Symptoms Scale (DSSS), SS somatic subscale of the DSSS, HADS-D depression subscale of the Hospital Anxiety and Depression Scale (HADS), HADS-A anxiety subscale of the HADS. Significance (*p* < 0.05): Group I *vs.* II: DS and SS; Group I *vs.* III: All five subscales; Group I *vs.* IV: All five subscales; Group II *vs.* III: HAMD, DS, and HADS-D; Group II *vs.* IV: All five subscales except for SS; Group III *vs.* IV: none.

Figure 1 shows the scores of depression, anxiety, and somatic symptoms in four subgroups. There was a trend that patients with a current MDE and migraine had the greatest severities of depression, anxiety, and somatic symptoms, followed by patients with a current MDE without migraine, patients with migraine without a current MDE, and patients without a current MDE or migraine, with the exception of the SS.

### Comparisons of the SF-36 subscales between patients with and without migraine

Table 4 shows the differences in the SF-36 subscales between patients with and without migraine. Patients with migraine had significantly (*p* < 0.006) lower scores (a poorer HRQoL) in the eight subscales as compared with patients without migraine. Similarly, patients with a current MDE also had significantly lower scores in all subscales. After controlling for demographic variables by a general linear model, the estimated differences between migraine *vs.* non-migraine and between a current MDE *vs.* no current MDE reached statistical significance and clinical significance (a difference in score > 5) in all eight subscales. Of note, the reduction attributed to migraine was slightly greater than that attributed to a current MDE in the PF (11.2 *vs.* 9.4) and BP (23.5 *vs.* 20.0) scores.

**Table 4:** Comparison of the Short-Form 36 subscales and headache indices between outpatients with and without migraine and MDE

	***Total (n = 214)***		***Yes***	***No***	***Estimated difference ***^**a**^
PF	80.1 ± 20.2	Migraine	71.9 ± 21.8**	84.5 ± 17.9	−11.2**
		MDE	74.4 ± 20.9**	84.7 ± 18.5	−9.4**
RP	39.7 ± 40.2	Migraine	23.7 ± 36.0**	48.4 ± 39.7	−23.7**
		MDE	24.7 ± 34.9**	51.7 ± 40.2	−28.0**
BP	55.1 ± 27.4	Migraine	38.3 ± 24.4**	64.2 ± 24.7	−23.5**
		MDE	43.6 ± 25.6**	64.4 ± 25.4	−20.0**
GH	39.3 ± 21.0	Migraine	31.3 ± 21.5**	43.6 ± 19.5	−11.6**
		MDE	29.7 ± 17.4**	46.9 ± 20.6	−15.9**
VT	31.1 ± 22.0	Migraine	21.6 ± 17.1**	36.3 ± 22.7	−13.2**
		MDE	17.5 ± 13.5**	42.0 ± 21.5	−23.6**
SF	50.9 ± 27.8	Migraine	39.5 ± 23.7**	57.0 ± 28.0	−18.8**
		MDE	36.6 ± 23.2**	62.3 ± 25.9	−25.7**
RE	26.5 ± 37.0	Migraine	11.6 ± 27.7**	34.5 ± 39.0	−22.8**
		MDE	6.0 ± 17.5**	42.9 ± 40.3	−35.4**
MH	34.7 ± 20.0	Migraine	26.8 ± 17.1**	39.0 ± 19.8	−10.9**
		MDE	22.7 ± 12.0**	44.3 ± 19.4	−20.5**
HI	3.3 ± 3.4	Migraine	6.0 ± 3.3**	1.9 ± 2.5	
		MDE	4.7 ± 3.4**	2.2 ± 3.0	
HF	2.6 ± 2.8	Migraine	4.6 ± 2.6**	1.6 ± 2.2	
		MDE	3.9 ± 2.8**	1.6 ± 2.2	

PF physical functioning, RP role limitations-physical, BP bodily pain, GH general health perceptions, VT vitality, SF social functioning, RE role limitations-emotional, MH mental health, HI average headache intensity in the past week, HF headache days in the past week, MDE a current major depressive episode.

^a^Estimated difference contributed by migraine or a MDE after controlling for five demographic variables. ** *p* < 0.006.

### Correlations of headache parameters to depression, anxiety, somatic severity, and the SF-36

Headache intensity was significantly correlated to the three psychometric scales (the correlation coefficient (*r*) ranged from 0.49 SS to 0.29 HADS-D, all *p* < 0.01) and the eight SF-36 subscales (*r*: 0.49 BP to 0.23 SF, all *p* < 0.01). The headache frequency was also significantly correlated to the three psychometric scales (*r*: 0.54 SS to 0.39 HADS-D, all *p* < 0.01) and all the SF-36 subscales (*r*: 0.47 BP to 0.28 RP, all *p* < 0.05).

### The impacts of migraine on depression, anxiety, and somatic symptoms

Table 5 identifies the factors that independently predict depression, anxiety, and somatic severities. After controlling for demographic variables and a current MDE, migraine was still an independent factor predicting the severities of depression, anxiety, and somatic symptoms. The variance of the SS explained by migraine was greater than that of a current MDE.

**Table 5:** Independent factors predicting depression, anxiety, somatic symptoms, and health-related quality of life among psychiatric outpatients

	***Independent variable***	***Beta***	***R***^***2 ***^***change***	***p value***
HAMD	MDE	0.58	0.44	< 0.01
	Being employed	−0.15	0.03	< 0.01
	Migraine	0.15	0.02	< 0.01
	Age	−0.11	0.01	0.03
DS	MDE	0.57	0.45	< 0.01
	Migraine	0.20	0.04	< 0.01
	Age	−0.12	0.01	0.01
	Gender	−0.11	0.01	0.03
SS	Migraine	0.32	0.23	< 0.01
	MDE	0.27	0.06	< 0.01
	Being employed	−0.15	0.04	0.01
	Gender	−0.15	0.02	0.02
HADS-D	MDE	0.57	0.39	< 0.01
	Migraine	0.16	0.03	0.01
	Being employed	−0.12	0.01	0.03
HADS-A	MDE	0.41	0.21	< 0.01
	Being employed	−0.13	0.02	0.03
	Migraine	0.14	0.02	0.04
PF	Migraine	−0.22	0.09	< 0.01
	MDE	−0.17	0.02	0.02
	Being employed	0.14	0.02	0.03
RP	MDE	−0.26	0.11	< 0.01
	Migraine	−0.20	0.03	< 0.01
BP	Migraine	−0.35	0.20	< 0.01
	MDE	−0.25	0.05	< 0.01
	Being employed	0.13	0.02	0.03
GH	MDE	−0.36	0.17	< 0.01
	Being employed	0.15	0.03	0.01
	Migraine	−0.13	0.02	0.045
VT	MDE	−0.55	0.31	< 0.01
	Being employed	0.17	0.03	< 0.01
SF	MDE	−0.40	0.21	< 0.01
	Migraine	−0.17	0.02	0.01
	Educational years	−0.15	0.02	0.01
RE	MDE	−0.42	0.25	< 0.01
	Age	0.15	0.02	0.01
	Being employed	0.12	0.02	0.04
	Migraine	−0.13	0.01	0.04
MH	MDE	−0.52	0.30	< 0.01
	Being employed	0.16	0.03	< 0.01
	Age	0.12	0.01	0.04

HAMD Hamilton Depression Rating Scale, DS depression subscale of the Depression and Somatic Symptoms Scale (DSSS), SS somatic subscale of the DSSS, HADS-D depression subscale of the Hospital Anxiety and Depression Scale (HADS), HADS-A anxiety subscale of the HADS, PF physical functioning, RP role limitations-physical, BP bodily pain, GH general health perceptions, VT vitality, SF social functioning, RE role limitations-emotional, MH mental health, MDE a current major depressive episode.

### The impacts of migraine on the SF-36 subscales

After controlling for demographic variables and a current MDE, migraine was still an independent factor predicting all the SF-36 subscales, except for VT and MH (Table 5). The variance in the PF and the BP explained by migraine was greater than that of a current MDE.

## Discussion

Our study showed that the presence of migraine among psychiatric outpatients was associated with a greater severity of depression, anxiety, and somatic symptoms, and a poorer HRQoL. Comorbidity with migraine explained a higher variance on the physical and pain symptoms compared to a current MDE. Depression and/or anxiety are often considered important factors related to the severity of somatic or pain symptoms; however, the role of migraine in somatic and pain symptoms in the psychiatric field has long been neglected. This study first pointed out that the importance of migraine was significantly greater than that of a MDE in predicting pain and somatic symptoms in the regression models.

Migraine might be a surrogate for somatic and pain symptoms among these patients. This is imperative because pain or somatic symptoms are common residual symptoms, which are related to a poorer prognosis [34], among depressive patients. It is possible that simultaneous prevention of migraine during treatment for mood and anxiety disorders might decrease residual somatic and pain symptoms and improve the prognosis. Therefore, physicians should integrate the treatment of migraine into psychiatric treatment plans. In fact, migraine was common (35.0%) among these psychiatric outpatients. Patients with bipolar II disorder had the highest percentage (70%) of migraine. This result is compatible with Fasmer’s report (77%) [19]. The percentage of migraine among patients with MDD was 49.1%, which was close to that reported in previous studies [14,19]. The percentage of migraine among patients with only anxiety disorder was 24.5%, which was lower than that reported in the study of Senaratne et al. (67%) in an anxiety disorders clinic [17]. This might partially result from our diagnostic algorithm, i.e., in patients with only anxiety disorder, other psychiatric comorbidities, such as MDD and bipolar disorders, were excluded.

Our results that psychiatric outpatients with migraine were associated with more somatic or pain symptoms might be explained by two possible reasons. 1) Imbalance of neurotransmitters, such as serotonin, noradrenalin, and dopamine, is responsible for the pathogenesis of migraine as well as somatic symptoms and depression [35,36]. This is commonly used to explain the close relationship between migraine, depression, and somatic symptoms; 2) Repeated headache attacks might cause a similar effect to central sensitization, which is associated with comorbid pain conditions and a worse headache-related disability and increases somatic or pain symptoms among psychiatric outpatients with migraine [37].

There are several points, which might be helpful for clinical practice, worth noting. 1) Headache intensity and frequency were significantly correlated to the severity of depression, anxiety, somatic symptoms, and HRQoL. Therefore, headache intensity and frequency could be a useful index to predict these mental symptoms and HRQoL. 2) Depression and migraine independently predicted negative impacts on quality of life in a general population [38]. Our results demonstrated that migraine and a current MDE were independently associated with depression, anxiety, somatic symptoms, and HRQoL among these psychiatric outpatients. 3) The study showed that patients with both a MDE and migraine had the greatest severity of depression, anxiety, and somatic symptoms. This demonstrated the synergic effect of depression and migraine. 4) Psychiatric outpatients with migraine had an increased risk of a suicide attempt history. This result was compatible with previous studies [39]. In fact, migraine has been neglected in terms of evaluating suicide risk among psychiatric outpatients.

Our study has the following limitations. 1) Our study enrolled psychiatric outpatients in a medical center with several exclusion criteria to prevent confounding effects. This enrollment process might introduce bias. For example, the study excluded patients with a current manic episode or with psychotic features. Therefore, this study did not enroll patients with bipolar I disorder. 2) The sample size of some diagnoses, such as bipolar II disorder, was small. The study design, mixing different diagnoses of mood and anxiety disorders, had the advantage of reflecting the percentage of migraine among global non-psychotic outpatients. 3) The headache intensity and frequency in the past week were recalled by patients. Collecting data on the two headache parameters prospectively by headache diary might be more reliable. However, withholding pharmacological treatment while prospectively observing headache parameters might result in ethical problems owing to the suicidal risk. 4) Depression and migraine are associated with an increased suicide risk. In the study, only the past history of suicide attempts was recorded. A comprehensive assessment of suicide risk was not performed.

## Conclusions

In conclusion, migraine was common among psychiatric outpatients with mood and/or anxiety disorders. Bipolar II disorder patients had the highest percentage of migraine, followed by MDD. Patients with migraine had a greater severity of depression, anxiety, and somatic symptoms, as well as a poorer HRQoL than those without. Of note, the association of migraine with PF, BP, and somatic symptoms might be greater than that of a current MDE. Because of the high frequency and significant impacts of migraine on physical and pain symptoms, future studies should explore how to integrate the treatment strategies of migraine, mood disorders and anxiety disorders.

## Competing interests

All authors declare that they have no competing interests.

## Authors’ contributions

SJW and CIH designed the study and wrote the protocol. CIH and CYL collected the data. SJW and CIH undertook the statistical analysis, and CIH wrote the first draft of the manuscript. All authors read and approved the final manuscript.
